# Comparison of metabolic and neurological comorbidities in Asian patients with psoriasis and atopic dermatitis

**DOI:** 10.1038/s41598-024-54407-z

**Published:** 2024-02-20

**Authors:** Hee Joo Yang, Mi Young Lee, Jeong Hyeon Lee, Chang Jin Jung, Woo Jin Lee, Chong Hyun Won, Mi Woo Lee, Joon Min Jung, Sung Eun Chang

**Affiliations:** 1 Department of Dermatology, Asan Medical Center, University of Ulsan College of Medicine, 88 Olympic-ro 43 gil, Songpa-gu, Seoul, 05505 Republic of Korea; 2 Asan Institute for Life Sciences, Asan Medical Center, Seoul, Republic of Korea

**Keywords:** Diseases, Risk factors

## Abstract

Although various comorbidities have been noted to be associated with atopic dermatitis (AD) and psoriasis, few studies have compared comorbidities between the two diseases, and little is known about whether these comorbidities vary by the subtypes of psoriasis. In this study of 1:1 age- and sex-matched pair analysis between patients diagnosed with either psoriasis or AD at Asan Medical Center between 1991 and 2020, comorbidities, as determined by the International Classification of Diseases-10 codes, and likelihood ratios of metabolic and neurologic comorbidities in psoriasis compared with AD were studied using a logistic regression model. Among a total of 14,128 patients, the psoriasis group had higher odds of obesity (odds ratio [95% confidence interval]: 1.49 [1.34–1.66]), hypertension (1.14 [1.03–1.26]), diabetes mellitus (1.46 [1.29–1.66]), chronic kidney disease (1.59 [1.22–2.08]), and Parkinson's disease (2.1 [1.15–3.83]) than the AD group. Subgroup analysis revealed that patients with plaque psoriasis had higher odds of obesity (1.18 [1.05–1.33]), hypertension (1.18 [1.06–1.32]), diabetes mellitus (1.53 [1.34–1.75]), chronic kidney disease (1.66 [1.26–2.17]), and Parkinson’s disease (2.12 [1.16–3.88]) compared with AD. Meanwhile, guttate psoriasis was associated with higher odds of dementia (3.63 [1.06–12.40]) and patients with generalized pustular psoriasis showed higher odds of diabetes mellitus (5.42 [1.56–18.83]) compared with AD. In conclusion, Asian patients with all types of psoriasis should be closely monitored for the development of metabolic and neurologic diseases, especially men and those aged ≥ 40 years.

## Introduction

The relationship between atopic dermatitis (AD) and psoriasis is unclear. The usual clinical presentation of AD involves ill-demarcated lesions with oozing, while psoriasis presents with sharply demarcated lesions with dry scales^1^. AD commonly manifests during childhood and resolves with age, whereas psoriasis usually appears during adolescence and early adulthood and does not improve with age. Pathologically, AD and psoriasis involve different subsets of T helper (Th) cells, with Th2 cells mediating AD and Th1 cells mediating psoriasis.

Similarities have been observed in the pathogenesis of AD and psoriasis. Both diseases are T-cell mediated chronic inflammatory diseases, with the intrinsic, Asian, and pediatric forms of AD showing greater similarity with psoriasis than the European–American form of AD, such as involvement of the interleukin (IL)-23/Th17 axis, supported by real-time PCR and immunohistochemistry of skin biopsy specimens in prior studies^2,3^. Genetic linkage analysis has revealed common susceptibility gene loci on chromosomes 1q21, 3q21, 17q25, and 20p12, which play roles in dermal inflammation and immune system activation in both AD and psoriasis^4^.

Comorbidities of psoriasis and AD have been of interest. As indicated by the term “atopic march,” AD is associated with allergic diseases^5^. Nonallergic diseases, including obesity, diabetes, and cardiovascular diseases, have also been reported as comorbidities of AD^6,7^. Similarly, psoriasis has been noted to be associated with various diseases, such as obesity, hypertension, diabetes, and cardiovascular diseases, in both adults and children^8,9^.

To date, studies have compared the comorbidities of psoriasis and AD in German and French populations^10–12^. Since Asian AD shows more similarities with psoriasis than Western AD, studies are needed to compare comorbidities in Korean patients with psoriasis and AD. The reported prevalence of AD in Koreans is 2.2% in all ages and 0.9% in adults aged over 18, while the prevalence of psoriasis in Koreans is 0.5% in all ages^13,14^. In addition, little is currently known about the comorbidities associated with different subtypes of psoriasis.

The objectives of this study were to compare the metabolic and neurological comorbidities in Asian patients with AD and psoriasis, and to evaluate the comorbidities associated with different subtypes of psoriasis.

## Methods

The database of the Asan Medical Center was searched to identify patients diagnosed with AD or psoriasis between January 1, 1991 and December 31, 2020. Psoriasis was defined by the presence of World Health Organization (WHO) International Classification of Diseases (ICD)-10 codes L401, L404, L405, L408, L409, L4000, and L4008, whereas AD was defined by the presence of ICD-10 codes L209, L272, L2084, L2085, and L2088. Patients with AD were matched 1:1 to patients with psoriasis by age and sex. The study design was approved by the Institutional Review Board of Asan Medical Center (2021-0512), which waived the requirement for informed consent due to the retrospective design of the study. All steps in the study were performed according to the relevant guidelines and regulations.

Demographic and clinical information was collected from patients’ medical records. Factors recorded included height, weight, abdominal circumference, systolic blood pressure (SBP), and diastolic blood pressure (DBP). Laboratory data included serum concentrations of the liver enzymes alkaline phosphatase (ALP), alanine aminotransferase (ALT), aspartate aminotransferase (AST), and gamma-glutamyl transferase (GGT); concentrations of total cholesterol, high-density lipoprotein (HDL), low-density lipoprotein (LDL), triglycerides (TG), fasting glucose, C-reactive protein (CRP), and immunoglobulin E (IgE); erythrocyte sedimentation rate (ESR); eosinophil counts; and glomerular filtration rate (GFR). The cutoff values of these laboratory tests are presented in Supplementary Table S1. Only clinical and laboratory data collected within 1 year of the initial diagnosis of AD or psoriasis were analyzed. When data were collected during different visits, those obtained nearest to the date of the initial diagnosis were included.

Obesity in the Korean population was defined as body mass index (BMI) ≥ 25 kg/m^2^, in accordance with the WHO guidelines for the Asia–Pacific region. The presence of other metabolic and neurological comorbidities between 1991 and 2020 in patients with AD or psoriasis was based on ICD-10 diagnostic codes, including those for dyslipidemia, hypertension, diabetes mellitus, arrhythmia, chronic kidney disease, atherosclerosis, dementia, and Parkinson’s disease (Supplementary Table S2). Patients’ visits to other medical specialists, including endocrinologists, cardiologists, nephrologists, and neurologists, were monitored to enhance the coding accuracy of their metabolic and neurological comorbidities.

Types of psoriasis were subclassified as plaque, guttate, erythrodermic, generalized pustular psoriasis (GPP), or psoriatic arthritis (PsA). Palmoplantar pustulosis was excluded, as its diagnosis is frequently confused with that of dyshidrotic eczema in medical records. Patients with erythrodermic psoriasis were excluded from the subgroup analysis, as the number of these patients was too small for statistical comparisons.

### Statistical analysis

Continuous data in the two groups were presented as mean ± standard deviation and compared using *t*-tests or Mann–Whitney tests. Categorical variables in the two groups were compared using chi-square tests or linear association tests. Comorbidities associated with AD and psoriasis were compared after adjusting for the number of visits using logistic regression modeling. Results are presented as odds ratios (ORs) with 95% confidence intervals (CIs). All statistical analyses were performed using R version 3.5.3 (R Foundation for Statistical Computing) software, with *p* < 0.05 considered statistically significant.

## Results

### Patient characteristics

Altogether, 40,156 AD patients and 8712 psoriasis patients were identified. Matching by sex and age resulted in 14,128 patients, 7064 diagnosed with psoriasis and 7064 diagnosed with AD (shown in Supplementary Fig. S1). Their demographic, clinical, and laboratory data are summarized in Table 1. Each group consisted of 3584 male and 3480 female patients with a mean age at the time of diagnosis of 38.4 years.

**Table 1:** Demographics, clinical, and laboratory data of the patients.

		Atopic dermatitis	Psoriasis	*p*-value
Sex (no., (%))	Female	3480 (49.3)	3480 (49.3)	
	Male	3584 (50.7)	3584 (50.7)	
Age, yr (mean ± SD)		38.4 ± 17.3	38.4 ± 17.3	
Number of visits (mean ± SD)		7.2 ± 24.0	17.6 ± 60.5	
Height, cm (mean ± SD)		162.1 ± 14.8	164.2 ± 10.9	
Weight, kg (mean ± SD)		61.8 ± 16.0	65.4 ± 14.7	
BMI (kg/m^2^, mean ± SD)		23.1 ± 4.1	24.2 ± 4.1	< 0.001*
Abdominal circumference (cm, mean ± SD)		81.0 ± 9.9	86.5 ± 10.3	< 0.001*
Systolic blood pressure	Normal (< 120 mmHg)	1711 (44.1)	870 (40.0)	0.002*
	High	2165 (55.9)	1303 (60.0)	
Diastolic blood pressure	Normal (< 80 mmHg)	2870 (74.0)	1457 (67.1)	< 0.001*
	High	1006 (26.0)	716 (32.9)	
Alkaline phosphatase (ALP)	Normal (40–120 IU/L)	4381 (80.3)	2765 (81.0)	0.36
	Abnormal	1078 (19.7)	647 (19.0)	
Alanine aminotransferase (ALT)	Normal (≤ 40 IU/L)	4945 (89.6)	2918 (84.8)	< 0.001*
	High	573 (10.4)	524 (15.2)	
Aspartate aminotransferase (AST)	Normal (≤ 40 IU/L)	5161 (93.6)	3031 (88.2)	< 0.001*
	High	351 (6.4)	407 (11.8)	
Gamma-glutamyl transferase (GGT)	Normal (8–61 IU/L for males, 5–36 IU/L for females)	1054 (82.6)	856 (73.9)	< 0.001*
	High	222 (17.4)	303 (26.1)	
Fasting glucose	Normal (< 100 mg/dL)	58 (43.3)	66 (30.8)	0.025*
	High	76 (56.7)	148 (69.2)	
Total cholesterol	Normal (< 200 mg/dL)	3990 (73.7)	2391 (70.0)	< 0.001*
	High	1423 (26.3)	1024 (30.0)	
High-density lipoprotein (HDL)	Normal (≥ 40 mg/dL)	1457 (80.7)	1158 (76.8)	0.007*
	Low	348 (19.3)	349 (23.2)	
Low-density lipoprotein (LDL)	Normal (< 130 mg/dL)	1001 (67.7)	675 (65.0)	0.171
	High	477 (32.3)	363 (35.0)	
Triglyceride (Tg)	Normal (< 200 mg/dL)	1644 (84.4)	1269 (80.6)	0.003*
	High	303 (15.6)	306 (19.4)	
C-reactive protein (CRP)	Normal	1393 (78.4)	710 (63.2)	< 0.001*
	High	384 (21.6)	413 (36.8)	
Erythrocyte sedimentation rate (ESR)	Normal (≤ 9 mm/h (male) or ≤ 20 mm/h (female))	1665 (65.6)	800 (50.0)	< 0.001*
	High	872 (34.4)	821 (50.0)	
Glomerular filtration rate (GFR)	Normal (≥ 60 mL/min/1.73 m^2^)	4309 (97.5)	2219 (94.0)	< 0.001*
	Abnormal	109 (2.5)	141 (6.0)	
Total IgE	Normal (< 100 KU/L)	592 (33.8)	99 (53.8)	< 0.001*
	High	1158 (66.2)	85 (46.2)	
Eosinophil counts	Normal (< 500/μL)	4530 (84.4)	3011 (92.5)	< 0.001*
	Abnormal	835 (15.6)	245 (7.5)	

SD, standard deviation.

*p-value < 0.05.

The psoriasis group had a significantly higher BMI (24.2 ± 4.0 kg/m^2^ vs. 23.1 ± 4.1 kg/m^2^; *p* < 0.001) and abdominal circumference (86.5 ± 10.3 cm vs. 81.0 ± 9.9 cm; *p* < 0.001) than the AD group. Abnormalities in SBP (60.0% [1303/2173] vs. 55.9% [2165/3876], *p* = 0.002) and DBP (32.9% [716/2173] vs. 26.0% [1006/3876], *p* < 0.001) were significantly more frequent in the psoriasis than the AD group. Abnormal concentrations of liver enzymes, including ALT (15.2% [524/3442] vs. 10.4% [573/5518], *p* < 0.001), AST (11.8% [407/3438] vs. 6.4% [351/5512], *p* < 0.001), and GGT (26.1% [303/1159] vs. 17.4% [222/1276], *p* < 0.001), were more frequently observed in patients with psoriasis than with AD. Abnormal concentrations of fasting glucose (69.2% [148/214] vs. 56.7% [76/134], *p* = 0.025), total cholesterol (30.0% [1024/3415] vs. 26.3% [1423/5413], *p* < 0.001), HDL (23.2% [349/1507] vs. 19.3% [348/1805], *p* = 0.007), and TG (19.4% [306/1575] vs. 15.6% [303/1947], *p* = 0.003) were also significantly more frequent in the psoriasis group compared with the AD group, but the percentages with abnormal LDL concentrations did not differ between these two groups. Abnormal increases in inflammatory markers, including CRP (36.8% [413/1123] vs. 21.6% [384/1777], *p* < 0.001) and ESR (50.0% [800/1600] vs. 34.4% [872/2537], *p* < 0.001), as well as abnormalities in GFR (6.1% [6.0% [141/2360] vs. 2.5% [109/4418], *p* < 0.001), were significantly more frequent in the psoriasis than in the AD group. In contrast, abnormalities in total IgE concentration (5.9% [85/185] vs. 66.2% [1158/1751], *p* < 0.001) and eosinophil counts (7.5% [245/3256] vs. 15.6% [835/5365], *p* < 0.001) were significantly less frequent in the psoriasis group compared with the AD group.

### Metabolic and neurological comorbidities in patients with psoriasis and atopic dermatitis

**Figure 1:**
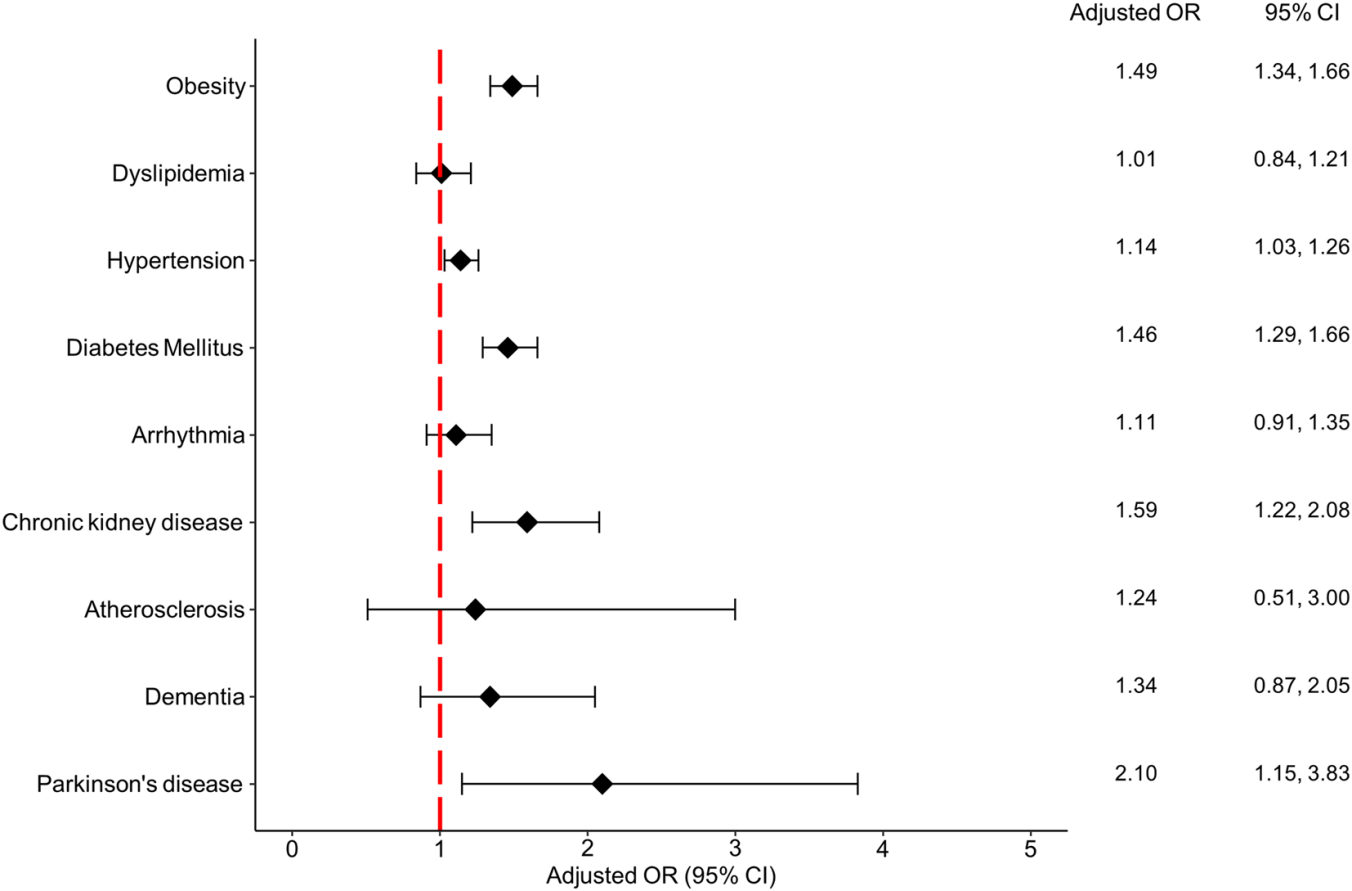
Forest plot of the adjusted odds of comorbidities in patients with psoriasis compared with age- and sex-matched patients with atopic dermatitis. OR, odds ratio; CI, confidence interval. Adjusted by the number of visits.

The adjusted ORs and 95% CIs of comorbidities in patients with psoriasis compared with age- and sex-matched patients with AD are summarized in Fig. 1. Relative to patients with AD, the adjusted ORs in psoriasis were 1.49 (95% CI 1.34–1.66) for obesity, 1.14 (95% CI 1.03–1.26) for hypertension, 1.46 (95% CI 1.29–1.66) for diabetes mellitus, 1.59 (95% CI 1.22–2.08) for chronic kidney disease, and 2.1 (95% CI 1.15–3.83) for Parkinson’s disease.

### Metabolic and neurological comorbidities in patients with psoriasis and atopic dermatitis according to sex

**Figure 2:**
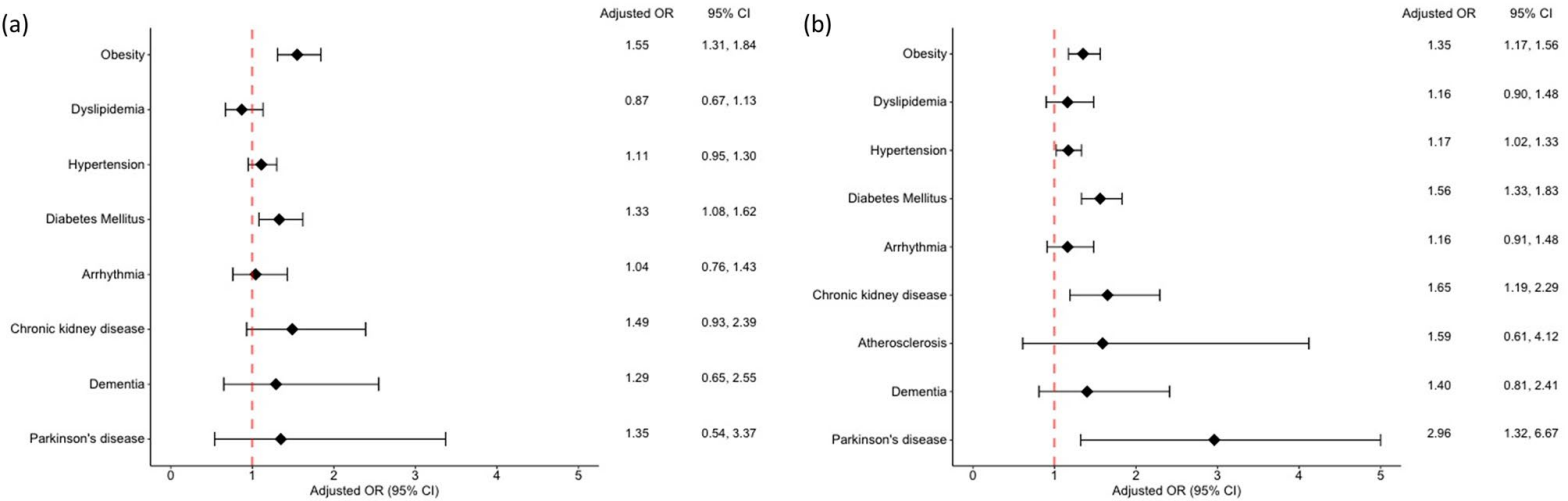
Forest plots of the adjusted odds of comorbidities in patients with psoriasis compared with age- and sex-matched patients with atopic dermatitis according to sex. (**a**) Female patients. (**b**) Male patients. OR, odds ratio; CI, confidence interval. Adjusted by the number of visits.

The adjusted ORs of comorbidities according to sex are shown in Fig. 2. Compared with women and men with AD, the adjusted ORs for women and men with psoriasis were 1.55 (95% CI 1.31–1.84) and 1.35 (95% CI 1.17–1.56), respectively, for obesity, and 1.33 (95% CI 1.08–1.62) and 1.56 (95% CI 1.33–1.83) for diabetes mellitus. The ORs for hypertension (1.17; 95% CI 1.02–1.33), chronic kidney disease (1.65; 95% CI 1.19–2.29), and Parkinson’s disease (2.96; 95% CI 1.32–6.67) were significantly higher in men, but not in women, with psoriasis relative to men with AD.

### Metabolic and neurological comorbidities in patients with psoriasis and atopic dermatitis according to age

**Figure 3:**
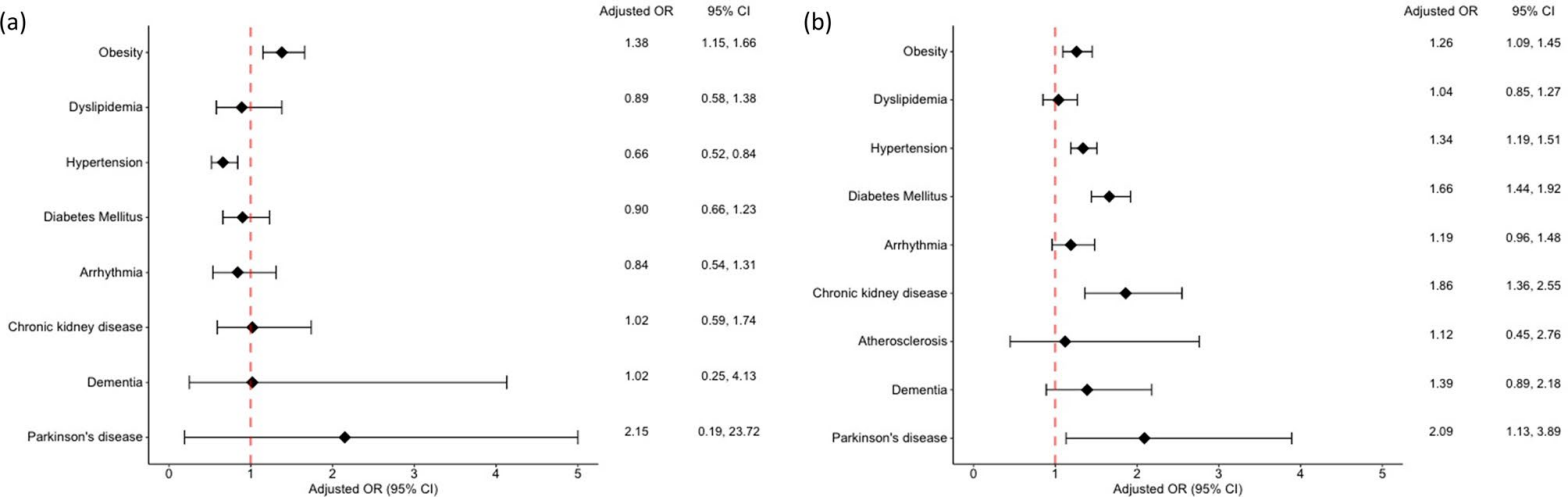
Forest plots of the adjusted odds of comorbidities in patients with psoriasis compared with age- and sex-matched patients with atopic dermatitis according to age group. (**a**) Patients aged < 40 years. (**b**) Patients aged ≥ 40 years. OR, odds ratio; CI, confidence interval. Adjusted by the number of visits.

The adjusted ORs of comorbidities according to age are shown in Fig. 3. Compared with age-matched patients with AD, the ORs for obesity were higher in patients with psoriasis aged < 40 (1.38; 95% CI 1.15–1.66) and ≥ 40 (1.26; 95% CI 1.09–1.45) years. Although the OR for hypertension was higher in psoriasis than in AD patients aged ≥ 40 years (1.34; 95% CI 1.19–1.51), the OR for hypertension was significantly lower in psoriasis patients than in AD patients aged < 40 years (0.66; 95% CI 0.52–0.84). The ORs for diabetes mellitus (1.66; 95% CI 1.44–1.92), chronic kidney disease (1.86; 95% CI 1.36–2.55), and Parkinson’s disease (2.09; 95% CI 1.13–3.89) were higher in psoriasis than in AD patients aged ≥ 40 years, but no statistically significant differences in these comorbidities were observed in psoriasis and AD patients aged < 40 years.

### Subgroup analysis according to the type of psoriasis

Of the 7064 psoriasis patients, 6504 were classified as having plaque psoriasis, 434 had guttate psoriasis, 103 had PsA, 19 had GPP, and four had erythrodermic psoriasis. The estimated ORs of comorbidities according to the subtype of psoriasis are presented in Table 2. In univariate logistic regression, unadjusted ORs for obesity (1.54; 95% CI 1.38–1.71), hypertension (1.20; 95% CI 1.08–1.33), diabetes mellitus (1.52; 95% CI 1.34–1.72), chronic kidney disease (1.70; 95% CI 1.30–2.22), and Parkinson’s disease (2.18; 95% CI 1.19–3.97) were significantly higher in patients with plaque psoriasis compared with patients with AD. For guttate psoriasis, ORs for dyslipidemia (0.39; 95% CI 0.17–0.88), hypertension (0.41; 95% CI 0.27–0.64), and diabetes mellitus (0.55; 95% CI 0.33–0.92) were lower compared with AD, whereas for generalized pustular psoriasis and PsA, ORs for diabetes mellitus were 3.86; 95% CI 1.28–11.69 and 2.09; 95% CI 1.16–3.77, respectively, compared with AD.

**Table 2:** Comorbidities according to psoriasis subtypes compared with atopic dermatitis.

Comorbidities	Group	Unadjusted OR (95% CI)	*p*-value	Adjusted OR (95% CI)	*p*-value
Obesity (BMI ≥ 25)	Atopic dermatitis	Reference			
	Plaque psoriasis	1.54 (1.38–1.71)	< 0.001*	1.18 (1.05–1.33)	0.0048*
	Guttate psoriasis	0.85 (0.51–1.45)	0.5583	0.87 (0.51–1.49)	0.6081
	Generalized pustular psoriasis	0.45 (0.10–2.03)	0.2992	0.49 (0.10–2.28)	0.3618
	Psoriatic arthritis	1.02 (0.60–1.75)	0.9371	0.85 (0.49–1.48)	0.5739
Dyslipidemia	Atopic dermatitis	Reference			
	Plaque psoriasis	1.08 (0.90–1.29)	0.4297	1.04 (0.86–1.25)	0.6978
	Guttate psoriasis	0.39 (0.17–0.88)	0.0237*	0.63 (0.28–1.43)	0.2675
	Generalized pustular psoriasis	3.27 (0.75–14.25)	0.1139	3.51 (0.76–16.13)	0.1073
	Psoriatic arthritis	0.27 (0.04–1.96)	0.1971	0.22 (0.03–1.56)	0.129
Hypertension	Atopic dermatitis	Reference			
	Plaque psoriasis	1.20 (1.08–1.33)	< 0.001*	1.18 (1.06–1.32)	0.0037*
	Guttate psoriasis	0.41 (0.27–0.64)	< 0.001*	0.80 (0.51–1.27)	0.3445
	Generalized pustular psoriasis	2.07 (0.69–6.25)	0.1969	2.61 (0.75–9.10)	0.1328
	Psoriatic arthritis	1.32 (0.76–2.30)	0.32	1.02 (0.57–1.82)	0.939
Diabetes mellitus	Atopic dermatitis	Reference			
	Plaque psoriasis	1.52 (1.34–1.72)	< 0.001*	1.53 (1.34–1.75)	< 0.001*
	Guttate psoriasis	0.55 (0.33–0.92)	0.023*	1.11 (0.65–1.88)	0.7054
	Generalized pustular psoriasis	3.86 (1.28–11.69)	0.0167*	5.42 (1.56–18.83)	0.0078*
	Psoriatic arthritis	2.09 (1.16–3.77)	0.014*	1.75 (0.95–3.24)	0.0716
Arrhythmia	Atopic dermatitis	Reference			
	Plaque psoriasis	1.16 (0.96–1.41)	0.1326	1.13 (0.93–1.38)	0.2206
	Guttate psoriasis	0.63 (0.31–1.29)	0.2065	1.19 (0.58–2.46)	0.6396
	Generalized pustular psoriasis	NE	NE	NE	NE
	Psoriatic arthritis	NE	NE	NE	NE
Chronic kidney disease	Atopic dermatitis	Reference			
	Plaque psoriasis	1.70 (1.30–2.22)	< 0.001*	1.66 (1.26–2.17)	0.0003*
	Guttate psoriasis	0.36 (0.09–1.48)	0.1572	0.65 (0.16–2.66)	0.5481
	Generalized pustular psoriasis	NE	NE	NE	NE
	Psoriatic arthritis	1.55 (0.38–6.39)	0.5427	1.31 (0.32–5.42)	0.7115
Atherosclerosis	Atopic dermatitis	Reference			
	Plaque psoriasis	1.33 (0.55–3.21)	0.5282	1.28 (0.53–3.10)	0.5864
	Guttate psoriasis	NE	NE	NE	NE
	Generalized pustular psoriasis	NE	NE	NE	NE
	Psoriatic arthritis	NE	NE	NE	NE
Dementia	Atopic dermatitis	Reference			
	Plaque psoriasis	1.38 (0.90–2.13)	0.1419	1.33 (0.86–2.07)	0.2012
	Guttate psoriasis	1.32 (0.41–4.30)	0.6431	3.63 (1.06–12.40)	0.0401*
	Generalized pustular psoriasis	NE	NE	NE	NE
	Psoriatic arthritis	1.86 (0.25–13.70)	0.5416	1.72 (0.23–12.94)	0.5961
Parkinson’s disease	Atopic dermatitis	Reference			
	Plaque psoriasis	2.18 (1.19–3.97)	0.0111*	2.12 (1.16–3.88)	0.0151*
	Guttate psoriasis	1.02 (0.13–7.69)	0.9867	2.60 (0.33–20.25)	0.3608
	Generalized pustular psoriasis	NE	NE	NE	NE
	Psoriatic arthritis	NE	NE	NE	NE

OR, odds ratio; CI, confidence interval; BMI, body mass index; NE, not estimated.

Adjusted by age, sex, and the number of visits.

*p-value < 0.05.

In multivariate logistic regression, patients with plaque psoriasis had higher adjusted ORs for obesity (1.18; 95% CI 1.05–1.33), hypertension (1.18; 95% CI 1.06–1.32), diabetes mellitus (1.53; 95% CI 1.34–1.75), chronic kidney disease (1.66; 95% CI 1.26–2.17), and Parkinson’s disease (2.12; 95% CI 1.16–3.88). Relative to patients with AD, patients with guttate psoriasis had a significantly higher OR for dementia (3.63; 95% CI 1.06–12.40) and patients with generalized pustular psoriasis had a significantly higher OR for diabetes mellitus (5.42; 95% CI 1.56–18.83).

## Discussion

Psoriasis and AD have each been reported to be independently associated with metabolic comorbidities relative to the general population^6–9,15^. Few reports to date have compared the metabolic comorbidities associated with psoriasis and AD, especially in Asian patients^10–12^. German studies showed that the prevalences of arterial hypertension, hyperlipidemia, obesity, and diabetes mellitus are higher in psoriasis patients than AD patients^10,11^. Similarly, the present study found that Asian patients with psoriasis had higher ORs for obesity, hypertension, diabetes, and chronic kidney disease than age- and sex-matched AD counterparts. These findings are supported by a high BMI and abdominal circumference, frequent abnormalities in blood pressure and fasting glucose, and low GFR levels in the psoriasis group compared with the AD group. Moreover, the significantly higher CRP concentrations and ESR in psoriasis patients suggest that systemic inflammation may play a role in the more frequent development of metabolic comorbidities in association with psoriasis. It may be noteworthy to point out that an age-matched comparator diagnosed with AD is more likely to have had the inflammatory skin disease longer than a patient with psoriasis, as AD usually begins earlier in the lifetime compared with psoriasis. However, the higher level of systemic inflammation in psoriasis supported by laboratory data shows that attention needs to be paid to inflammatory comorbidities in patients with psoriasis.

The prevalence of obesity, dyslipidemia, hypertension, diabetes mellitus, arrhythmia, CKD, atherosclerosis, dementia, and Parkinson’s disease were as follows: obesity 36.3% (all ages)^16^, dyslipidemia 19.9% (all ages)^17^, hypertension 29.4% (aged 20 or over)^18^, diabetes mellitus 13.9% (aged 20 or over)^19^, arrhythmia 15% (aged 20 or over)^20^, CKD 5% (aged 35 or over)^21^, atherosclerosis 10% (aged 18 or over)^22^, dementia 6.9% (aged 60 or over)^23^, and Parkinson’s disease 0.4% (aged 50 or over)^24^. The ORs for dyslipidemia were previously shown to be higher in patients with both psoriasis^25^ and AD^26^ compared with the general population. Although the present study found that the prevalence of dyslipidemia, as determined by ICD-10 codes, did not differ significantly between the psoriasis and AD groups, the lipid profiles, including concentrations of HDL, TGs, and total cholesterol, differed significantly between these two groups, suggesting that dyslipidemia may be relatively underdiagnosed in psoriasis patients compared with AD patients.

In agreement with previous findings, the present study found that AD patients were more likely to have concurrent eosinophilia and high serum IgE levels than psoriasis patients^27,28^. Patients with AD more frequently present with elevated serum total IgE levels and peripheral eosinophilia than those without AD, with the levels of both correlating significantly with AD severity, as measured by the Eczema Areas and Severity Index scores^28^.

Regarding sex-related comorbidity statistics compared to the general population, recent reports from the United States and Germany showed that the associations of psoriasis with metabolic and cardiovascular risk factors were stronger in women than in men^29,30^. In contrast, according to Taiwanese administrative data, the prevalences of metabolic comorbidities, including hypertension, dyslipidemia, and diabetes mellitus, as well as liver and renal diseases, were significantly higher in men with psoriasis than in women with psoriasis^31^. Previous studies that compared comorbidities of psoriasis and AD did not provide data according to sex^10–12^. In this Korean study, the odds for obesity and diabetes mellitus were higher in both men and women with psoriasis than with AD, but only men with psoriasis showed higher odds of hypertension, chronic kidney disease, and Parkinson’s disease than men with AD. These discrepancies may be due to differences in ethnicity and possibly lifestyles and dietary styles.

Regarding age-related comorbidities compared to the general population, a recent meta-analysis showed that children with psoriasis had high pooled ORs for obesity, hypertension, diabetes, dyslipidemia, and metabolic syndrome^32^. In this study, the ORs for obesity were higher in people with psoriasis than AD, regardless of age. However, the OR for hypertension was lower in psoriasis patients aged < 40 years, but higher in those aged ≥ 40 years than in age-matched AD counterparts. While systemic medications including steroids and cyclosporine may play a role, a previous case–control study conducted in the US, which included patients aged under 18, showed that AD was associated with high odds of SBP even after adjusting for the use of steroids and cyclosporine^33^. In addition, despite the age-matching of patients in the present study, because AD begins at an earlier age than psoriasis, the disease duration before the first diagnosis may have been longer in the AD group, resulting in a higher OR for hypertension for AD patients than in those with psoriasis.

Our present study is interesting in showing that the likelihood of Parkinson’s disease was higher in patients with psoriasis than in those with AD. No link between AD and Parkinson’s disease has been reported before. Analyses of nationwide population-based cohort data from Taiwan and Korea have noted that the hazard ratio of Parkinson’s disease was significantly higher in psoriasis patients than in a control group^34,35^. These associations were presumed to be due to chronic inflammation, including the enhanced expression of proinflammatory cytokines such as tumor necrosis factor-α, IL-1, and IL-6, with attenuation of inflammation through systemic treatment reducing the risk of Parkinson’s disease^34^. Immune responses that promote T cell differentiation into Th17 cells, as well as the expression of genes such as *SETD1A* and *BC010367*, have been observed in both diseases^36,37^. In this study, the likelihood of Parkinson’s disease was higher in the psoriasis group, especially in men and patients aged 40 or over.

To our knowledge, little is known about the comorbidities of guttate psoriasis, probably due to the high rate of self-remission^38^. However, persistent cases of guttate psoriasis and conversion to plaque psoriasis had been reported in about 15–40%^39,40^. Consistent with our assumption that that odds ratio of inflammatory comorbidities of guttate psoriasis may be high, we found that the odds of dementia were higher than patients with atopic dermatitis in the present study. A recent meta-analysis revealed statistically significant associations between non-vascular dementia and psoriasis with a risk ratio of 1.13 (95% CI 1.11–1.15) and vascular dementia and psoriasis with a risk ratio of 1.41 (95% CI 1.09–1.82)^41^. Apolipoprotein E and IL-23/IL-17 may link psoriasis and Alzheimer’s dementia, the most common non-vascular dementia^42,43^. Arterial stiffness and increased levels of inflammation and oxidative stress may explain the link between psoriasis and vascular dementia^44,45^. Although serum levels of IL-2, IL-23, interferon-γ, and LL37 were found to be elevated in patients with psoriasis, there were no differences between plaque and guttate psoriasis^46^. The early age of onset of guttate psoriasis may lead to a long exposure to inflammatory cytokines. Also, streptococcal infection in guttate psoriasis may cause indirect neuronal damage through neuroinflammation, although the pathogenesis of streptococcal infection passing through the blood brain barrier needs to be elucidated^47,48^.

Meanwhile, a previous study on GPP reported obesity (42.9%), hypertension (25.7%), hyperlipidemia (25.7%), and diabetes mellitus (23.7%) as associated comorbidities^49^. Mutations in *IL36RN* and *CARD14*, which are linked to the upregulation of pro-inflammatory cytokines such as IL-1, have been identified in some GPP patients^50^. Higher expression of neutrophil chemokines, including CXCL1, CXCL2, and CXCL8, were observed in GPP patients compared with plaque psoriasis patients^51^. As the pathophysiology of psoriasis is viewed as a spectrum between autoimmunity and autoinflammation of which GPP more involves autoinflammation axis with high expression of inflammatory cytokines and chemokines, we assumed that the odds ratio of inflammatory comorbidities might be higher in pustular psoriasis group^52^. Consistently in the present study, higher odds of diabetes mellitus in the GPP group were found compared with plaque psoriasis group.

Although PsA was reported to be associated with higher odds of metabolic syndrome (OR 1.78; 95% CI 1.08–2.95) and renal function impairment as well as a high risk ratio of dementia (2.20; 95% CI 1.29–3.78) than patients with psoriasis alone, the present study did not find significant associations^41,53,54^. This may be due to the small number of patients with PsA included in this study, consistent with the low prevalence of PsA in Korea^14^.

The strengths of our study include the extensive amount of laboratory data, which support the findings on metabolic and neurologic comorbidities. Also, compared with previous studies that were designed to use administrative data and included diagnostic codes applied by nonspecialists, this study included hospital-recorded real clinical-based data only of patients who were diagnosed by dermatologists. Moreover, we report statistically significant ORs for metabolic and neurologic comorbidities in specific psoriasis subtypes, including guttate psoriasis and GPP.

This study also has several limitations. First, the comorbidities were based on diagnostic codes, making the accuracy of the data highly dependent on accurate coding by physicians in practice. To improve the accuracy, we checked whether the patients visited specialists for specific comorbidities. Second, this study included only those patients who visited a tertiary center for psoriasis and AD. Patients with mild disease who did not present at a tertiary center may therefore have been overlooked. Normal controls were not available since healthy individuals visiting dermatologic departments do not routinely undergo blood tests. Moreover, the comorbidities of interest were not subclassified according to their pathophysiology, limiting the interpretation of the study results.

In conclusion, Asian patients with all types of psoriasis should be closely monitored for the development of metabolic and neurologic diseases, especially men and those aged ≥ 40 years.

### Supplementary Information

Supplementary Information.

## Data Availability

All data generated or analysed during this study are included in this published article and supplementary information file. Additional data will be provided by the corresponding author when requested.
