# Supplementary material for: Comparison of metabolic and neurological comorbidities in Asian patients with psoriasis and atopic dermatitis
Source: Sci Rep. 2024 Feb 20;14:4212. doi: 10.1038/s41598-024-54407-z (PMC10879488; doi:10.1038/s41598-024-54407-z)
Supplement: Supplementary file 1 — Supplementary Information. [file 41598_2024_54407_MOESM1_ESM.pdf]

Supplementary Information

Hee Joo Yang, Mi Young Lee, Jeong Hyeon Lee, Chang Jin Jung, Woo Jin Lee, Chong Hyun Won, Mi Woo Lee, Joon Min Jung*, Sung Eun Chang*

Comparison of metabolic and neurological comorbidities in Asian patients with psoriasis and atopic dermatitis

Scientific Reports.

Figure S1. Study design flowchart

Table S1. Normal range for each laboratory test

Table S2. International Classification of Diseases-10 codes for each comorbidity

This supplementary material has been provided by the authors to give readers additional information about their work.

Figure S1. Study design flowchart

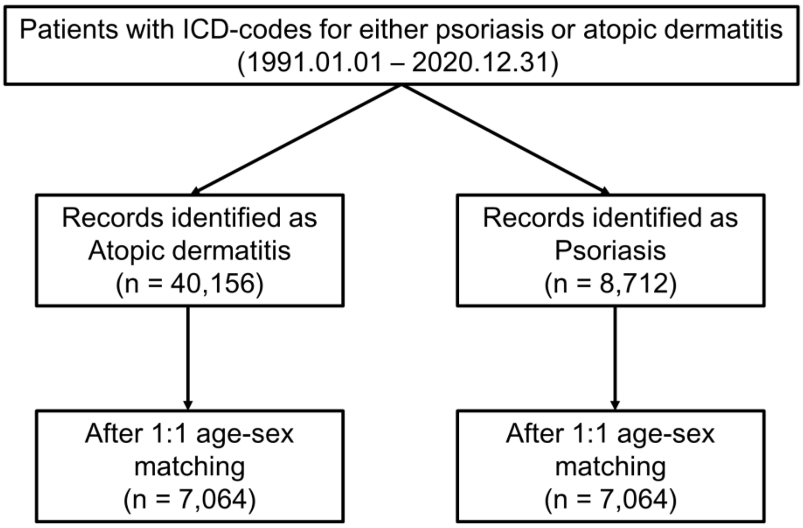

Abbreviations: ICD, International Classification of Diseases.

Table S1. Normal range for each laboratory test

Laboratory test	Normal range
Systolic Blood Pressure	< 120mmHg
Diastolic Blood Pressure	< 80mmHg
Alkaline phosphatase (ALP)	40-120 IU/L
Alanine aminotransferase (ALT)	≤ 40 IU/L
Aspartate aminotransferase (AST)	≤ 40 IU/L
Gamma-glutamyl transferase (GGT)	8-61 IU/L (male), 5-36IU/L (female)
Total cholesterol	< 200 mg/dL
High-density lipoprotein (HDL)	≥ 40 mg/dL
Low-density lipoprotein (LDL)	< 130 mg/dL
Triglyceride (Tg)	< 200 mg/dL
Fasting glucose	< 100 mg/dL
C-reactive protein (CRP)	< 0.6 mg/dL
Erythrocyte sedimentation rate (ESR)	≤ 9mm/h (male), ≤ 20mm/h (female)
Total IgE	< 100 KU/L
Eosinophil counts	< 500/µL
Glomerular filtration rate (GFR)	≥ 60 mL/min/1.73m^2

Abbreviations: IU, International Unit; KU, kilounit.

Table S2. International Classification of Diseases-10 codes for each comorbidity

Comorbidities	ICD codes
Dyslipidemia	E789, E1168, E7808, E782, E784, E785
Diabetes mellitus	E1010, E1028, E1031, E1032, E1033, E1034, E1038, E1040, E1041, E1048, I1071, I1072, E108, E109, E1100, E1101, E1108, E1110, E1120, E1121, E1122, E1128, E1131, E1132, E1133, E1134, E1138, E1141, E1142, E1148, E1150, E1151, E1164, E1165, E1168, E1170, E1171, E1172, E1178, E118, E119, E129, E1328, E138, E139, E1400, E1401, E1428. E1432, E1433, E1448, E1461, E1464, E1468, E1470, E1472, E148, E149, G590. G632, G990 H360, I792, K938 and N083
Arrhythmia	I4988, I499
Chronic kidney disease	E 1122, N185, T825
Atherosclerosis	I652, I7000, I7010, I7022, I7024, I7029, I70820, I70830, I70880, I70990
Dementia	F000, F001, F002, F009, F010, F011, F012, F013 F018, F019, F020, F028, F03, F050, F107, G1228, G238, G300, G301, G308, G309, G3100, G3182
Parkinson’s disease	F023, F067, F069, G20, G210, G211, G214, G219
